# Phthalic Acid Esters in Soils from Vegetable Greenhouses in Shandong Peninsula, East China

**DOI:** 10.1371/journal.pone.0095701

**Published:** 2014-04-18

**Authors:** Chao Chai, Hongzhen Cheng, Wei Ge, Dong Ma, Yanxi Shi

**Affiliations:** 1 College of Resources and Environment, Qingdao Agricultural University, Qingdao, China; 2 College of Life Sciences, Qingdao Agricultural University, Qingdao, China; Institute for Plant Protection (IPP), CNR, Italy

## Abstract

Soils at depths of 0 cm to 10 cm, 10 cm to 20 cm, and 20 cm to 40 cm from 37 vegetable greenhouses in Shandong Peninsula, East China, were collected, and 16 phthalic acid esters (PAEs) were detected using gas chromatography-mass spectrometry (GC-MS). All 16 PAEs could be detected in soils from vegetable greenhouses. The total of 16 PAEs (Σ_16_PAEs) ranged from 1.939 mg/kg to 35.442 mg/kg, with an average of 6.748 mg/kg. Among four areas, including Qingdao, Weihai, Weifang, and Yantai, the average and maximum concentrations of Σ_16_PAEs in soils at depths of 0 cm to 10 cm appeared in Weifang, which has a long history of vegetable production and is famous for extensive greenhouse cultivation. Despite the different concentrations of Σ_16_PAEs, the PAE compositions were comparable. Among the 16 PAEs, di(2-ethylhexyl) phthalate (DEHP), di-n-octyl phthalate (DnOP), di-n-butyl phthalate (DnBP), and diisobutyl phthalate (DiBP) were the most abundant. Compared with the results on agricultural soils in China, soils that are being used or were used for vegetable greenhouses had higher PAE concentrations. Among PAEs, dimethyl phthalate (DMP), diethyl phthalate (DEP) and DnBP exceeded soil allowable concentrations (in US) in more than 90% of the samples, and DnOP in more than 20%. Shandong Peninsula has the highest PAE contents, which suggests that this area is severely contaminated by PAEs.

## Introduction

Phthalic acid esters (PAEs) are used extensively as plasticizers of plastic products, such as polyvinyl chloride, and as nonplasticizers in consumer products, including medical devices, building materials, paints, pesticides, fertilizes, food packaging, and so on [1]. The large-scale production and application of 6.0 million tons/yr [2] of PAEs have made these materials ubiquitous environment pollutants [3]–[8]. Some PAEs have endocrine disruptive effects [9], and six PAEs are categorized as priority environmental pollutants by the United States Environmental Protection Agency [10].

Greenhouse cultivation has expanded dramatically in China since the 1980s, reaching up to 3.5 million ha by 2011 [11]. Greenhouse cultivation is mainly for vegetable production in China, and plastic greenhouses account for more than 99% of greenhouse cultivation relative to glass greenhouses [12]–[13]. Several studies detected PAEs in soils of vegetable greenhouses in Nanjing and Hangzhou [14]–[15], as well as in other agricultural soils, such as vegetable soils in Guangzhou and paddy soils in Leizhou Peninsula in China [16]–[17]. The buildup of PAEs in agricultural soils may contaminate agricultural products, and further raise the human health risk [18].

Shandong Peninsula is the largest Peninsula in China with rapid urbanization and high population density of 550 people/km^2^. The Peninsula includes the cities of Qingdao, Yantai, Weifang, and Weihai. Shandong Peninsula has a long history of vegetable greenhouse cultivation and is a main vegetable-producing region, with its greenhouse coverage accounting for approximately 50% of that of China. The vegetable greenhouses in this peninsula are close to the highly populated urban areas, and plastic film is widely used. Plastic film of 30000 tons/yr is estimated to be used only in one county, i.e., Shouguang in Weifang of Shandong Peninsula [15]. PAEs account for 10 wt% to 60 wt% of plastic products [9], [19], thus giving rise to concerns about the potential risk of PAEs in recent years. However, few studies focused on the characteristics of PAEs in soils of vegetable greenhouses in Shandong Peninsula.

This study provides information on the concentrations, compositions, and distributions of 16 PAEs in soils from vegetable greenhouses in Shandong Peninsula and discusses possible sources, influence factors, and potential environment risk.

## Materials and Methods

### Chemicals and materials

Mixed standard solutions of 16 PAEs containing dimethyl phthalate (DMP), diethyl phthalate (DEP), diisobutyl phthalate (DiBP), di-n-butyl phthalate (DnBP), dimethylglycol phthalate (DMGP), di(4-methyl-2-pentyl) phthalate (DMPP), di(2-ethylhexyl) phthalate (DEHP), di(2-ethoxyethyl) phthalate (DEEP), dipentyl phthalate (DPP), di-n-hexyl phthalate (DHXP), butylbenzyl phthalate (BBP), di(2-n-butoxyethyl) phthalate (DBEP), dicyclohexyl phthalate (DCHP), di-n-octyl phthalate (DnOP), diphenyl phthalate (DPhP), and di-n-nonyl phthalate (DNP) were supplied by O2SI, Inc. (USA). The concentration of each PAE in this mixture solution was 1000 mg/L. Glassware was steeped with K_2_CrO_7_/H_2_SO_4_ solution for 12 h, washed with redistilled water, and then baked at 300°C for 4 h. Acetone, petroleum ether, and diethyl ether were of analytical grade and re-distilled before use to avoid PAEs contamination. Hexane was of HPLC grade and purchased from Anpel Company Inc. Florisil (60 mesh to 80 mesh) was activated at 650°C, and anhydrous sodium sulfate was baked at 420°C for 4 h.

### Sampling

**Figure 1:**
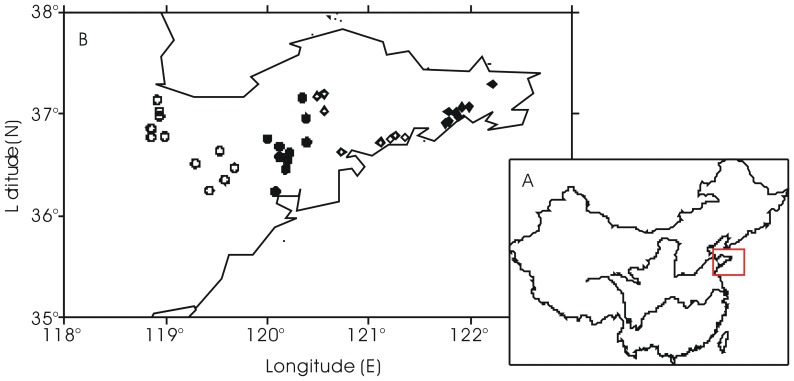
Schematic map showing the geographical location of (A) Shandong Peninsula and (B) the vegetable soil sampling sites in 4 regions in the Shandong Peninsula (solid round: Qingdao; solid diamond: Weihai; circle: Weifang; diamond: Yantai).

No specific permissions were required for sampling locations/activities. The field studies did not involve endangered or protected species. A total of 111 soil samples were collected from 37 vegetable greenhouses in Qingdao (number of samples: 30), Weihai (number of samples: 24), Weifang (number of samples: 33), and Yantai (number of samples: 24) in Shandong Peninsula in from 28 to 30 May 2012. The sampling locations are shown in Fig. 1.

Each sampling site consisted of five sub-samples (0.2 kg each) in the middle and four corners at depths of 0 cm to 10 cm, 10 cm to 20 cm, and 20 cm to 40 cm. The five sub-samples were mixed immediately after sampling, and then the soils were collected using aluminum foil envelopes through a pre-cleaned stainless steel auger and transported to laboratory in an ice box. Soils were stored in glass bottles at −20°C until analysis after being freeze-dried, ground, and homogenized with a stainless steel sieve (60 mesh). PAE contamination was avoided during sampling and further processing.

### Soil physical and chemical analyses

Soil pH was measured using a pH meter with a soil/water ratio of 1∶2.5. Soil cation exchange capacity (CEC) was analyzed using the Ba^2+^ compulsive exchange method [20]. Particle-size fraction was determined using the pipette method, and the soil texture was classified according to the Soil Survey Division Staff [21]. Total organic carbon (TOC) was determined using the wet oxidation method with chromate [22] and total nitrogen (TN) using micro-Kjeldahl digestion method [23].

### Sample extraction of PAEs and instrumental analysis

PAEs extraction was conducted according to Wang's methods [24]. 5.0 g soil was spiked with surrogate standard (benzyl benzoate) and extracted through ultrasonication for 15 min thrice with 90 mL of acetone/petroleum ether (1∶3, v∶v). The extracts were combined, filtered, and concentrated to approximately 1 mL. The extracts were cleaned with anhydrous sodium sulfate (3 g), florisil (6 g), and anhydrous sodium sulfate (3 g) on a glass column (1 cm i.d.). The column was washed with 10 mL of petroleum ether/diethyl ether (10∶0.4, v∶v), and then PAEs were eluted with 90 mL of petroleum ether/diethyl ether (10∶3, v∶v). The extracts were reduced to 1.0 mL in hexane, and internal standard (diisophenyl phthalate) was added before instrumental analysis.

Instrumental analysis was performed on an Agilent 6890 GC-5973 MSD gas chromatography-mass spectrometry system (GC-MS) in electron impact and selective ion monitoring modes according to Zeng et al. [25]. The GC column used was DB-5MS capillary column (30 m×0.25 mm i.d. ×0.25 mm film thickness, J&W Scientific). The column temperature program was 80°C (1 min), to 180°C (10°C/min, 1 min), to 300°C (2°C/min, 10 min). The carrier gas was helium with flow rate of 0.8 mL/min. Then, 1 µL of the extracts was injected into GC-MS in splitless injection mode, and the injector temperature was 250°C. The GC-MS transfer line was 280°C, and the post run temperature was 285°C for 2 min.

### Quality control and quality assurance

Quality assurance was performed by analyzing a procedural and solvent blank, a spiked blank every 10 samples, surrogate standards for each sample, and sample duplicate. DiBP, DnBP, and DEHP were subtracted from those in the soil samples because of the small amount in procedural blanks. The surrogate recoveries were 84.1%±8.5%, and no surrogate corrections were made to the final PAE concentrations reported. The calibration curves were used with six concentration levels of a standard mixture for PAEs quantitation. The standard mixture was analyzed every 10 samples to determine instrument stability and to confirm the calibration curve. The instrumental detection limits ranged from 1∼9 pg, calculated by signal to noise ratio of 10. The method detection limits for PAEs were determined as mean field blanks plus three times the standard deviation of the field blanks [25], ranging from 0.002 mg/kg for DEP to 0.024 mg/kg for DEHP.

## Results

### Properties of the soils from vegetable greenhouses

The major characteristics of soils from vegetable greenhouses in Shandong Peninsula are presented in Table 1. The pH (H_2_O) of soils was neutral in three sample areas, but was less than 6.5 in Weihai, which was moderately acidic. The TOC ranged from 19.3 g/kg to 32.2 g/kg and presented a decreasing trend with soil depth. The C/N ratio was approximately 20 to 30, except in Weifang, which had a value of more than 40 on average. This value indicated that the organic matter content was more C-rich. The C/N ratio presented an increasing trend with soil depth. The CEC followed a similar pattern as pH, with lower values in soil samples from Weihai. Most of the soils were sandy loam, and some were loam.

**Table 1:** The main characteristics of the soils from vegetable greenhouses in Shandong Peninsula.

Area	Soil depth (cm)	pH	TOC (g/kg)	TN (g/kg)	C/N	CEC (mol/kg)	Sand (%)	Silt (%)	Clay (%)
Qingdao	0∼10	6.62±0.64	31.7±9.8	1.3±0.4	26.44±0.90	0.14±0.05	54.1±3.8	27.6±6.6	17.8±3.7
	10∼20	6.52±0.56	29.6±13.3	1.1±0.3	26.02±0.36	0.15±0.06	53.2±5.4	25.9±4.6	15.5±4.6
	20∼40	6.64±0.42	25.1±7.1	0.8±0.3	33.49±9.12	0.11±0.05	46.7±4.6	24.6±7.8	15.9±4.2
Weihai	0∼10	6.31±0.56	30.0±3.8	1.4±0.6	23.89±6.93	0.07±0.03	50.7±3.4	22.1±0.7	17.1±2.7
	10∼20	6.10±0.43	27.7±4.1	1.0±0.1	27.33±2.72	0.09±0.01	55.3±6.2	29.6±9.9	15.6±1.4
	20∼40	5.90±0.55	19.3±7.2	0.7±0.2	27.32±9.13	0.08±0.04	52.6±4.5	33.2±1.6	16.0±2.5
Weifang	0∼10	6.88±0.46	32.2±6.0	1.7±3.1	40.49±5.39	0.13±0.09	61.3±0.2	37.8±7.7	19.9±6.0
	10∼20	6.96±0.36	27.9±12.1	0.9±0.9	41.81±8.63	0.13±0.06	57.0±9.8	33.6±2.6	17.9±5.6
	20∼40	6.99±0.49	23.4±4.3	1.2±2.3	42.55±5.78	0.14±0.08	59.2±2.1	28.1±5.9	21.1±7.4
Yantai	0∼10	6.46±0.66	24.0±6.4	1.3±0.4	18.95±5.92	0.10±0.02	57.8±3.4	38.7±9.5	18.9±4.6
	10∼20	6.56±0.96	23.7±6.3	1.1±0.4	22.99±5.90	0.10±0.03	57.4±4.2	38.6±4.4	18.0±3.0
	20∼40	7.10±0.99	20.5±5.0	0.7±0.1	29.22±9.46	0.11±0.03	55.1±6.1	33.6±6.6	16.3±1.7

### PAE concentrations in soils from vegetable greenhouses in Shandong Peninsula

All 16 PAEs were detected in soils from vegetable greenhouses (Table 2). Among them, three PAEs, namely, DEP, DnBP, and DEHP, were detected in all samples. The detection rates of another three PAEs (DMP, DiBP, and DnOP) were more than 90%. By contrast, the detection rates of DBEP and DNP were lower than 20%. The mean concentrations of DiBP, DnBP, DEHP, and DnOP were more than 1 mg/kg, higher than other PAEs. On the whole, the mean was almost systematically inferior to standard deviation, suggesting a very high heterogeneity between soils. The total of 16 PAEs (Σ_16_PAEs) in Shandong Peninsula ranged from 1.939 mg/kg to 35.442 mg/kg, with an average of 6.748 mg/kg.

**Table 2:** The detection rate and concentration of PAEs in all soil samples from vegetable greenhouses in Shandong Peninsula (n = 111).

PAEs	Detection rate (%)	Mean (mg/kg)	SD (mg/kg)	Minimum (mg/kg)	Maximum (mg/kg)
DMP	99.1	0.364	0.276	ND	1.245
DEP	100	0.108	0.169	0.002	1.051
DiBP	96.4	1.118	1.928	ND	11.434
DnBP	100	1.471	2.715	0.016	15.722
DMGP	23.4	0.015	0.031	ND	0.170
DMPP	48.6	0.246	0.405	ND	1.971
DEHP	100	1.465	1.207	0.073	5.323
DEEP	23.4	0.041	0.243	ND	2.556
DPP	58.6	0.088	0.098	ND	0.516
DHXP	64.9	0.084	0.157	ND	1.448
BBP	86.5	0.194	0.557	ND	5.691
DBEP	18.9	0.015	0.038	ND	0.267
DCHP	44.1	0.035	0.048	ND	0.204
DnOP	97.3	1.239	1.796	ND	14.397
DPhP	82.0	0.240	0.290	ND	2.371
DNP	19.8	0.026	0.060	ND	0.251
Σ_16_PAEs		6.748	5.716	1.939	35.442

ND: not detected. The data labeled as “ND” were treated as zero in further statistical treatment.

**Figure 2:**
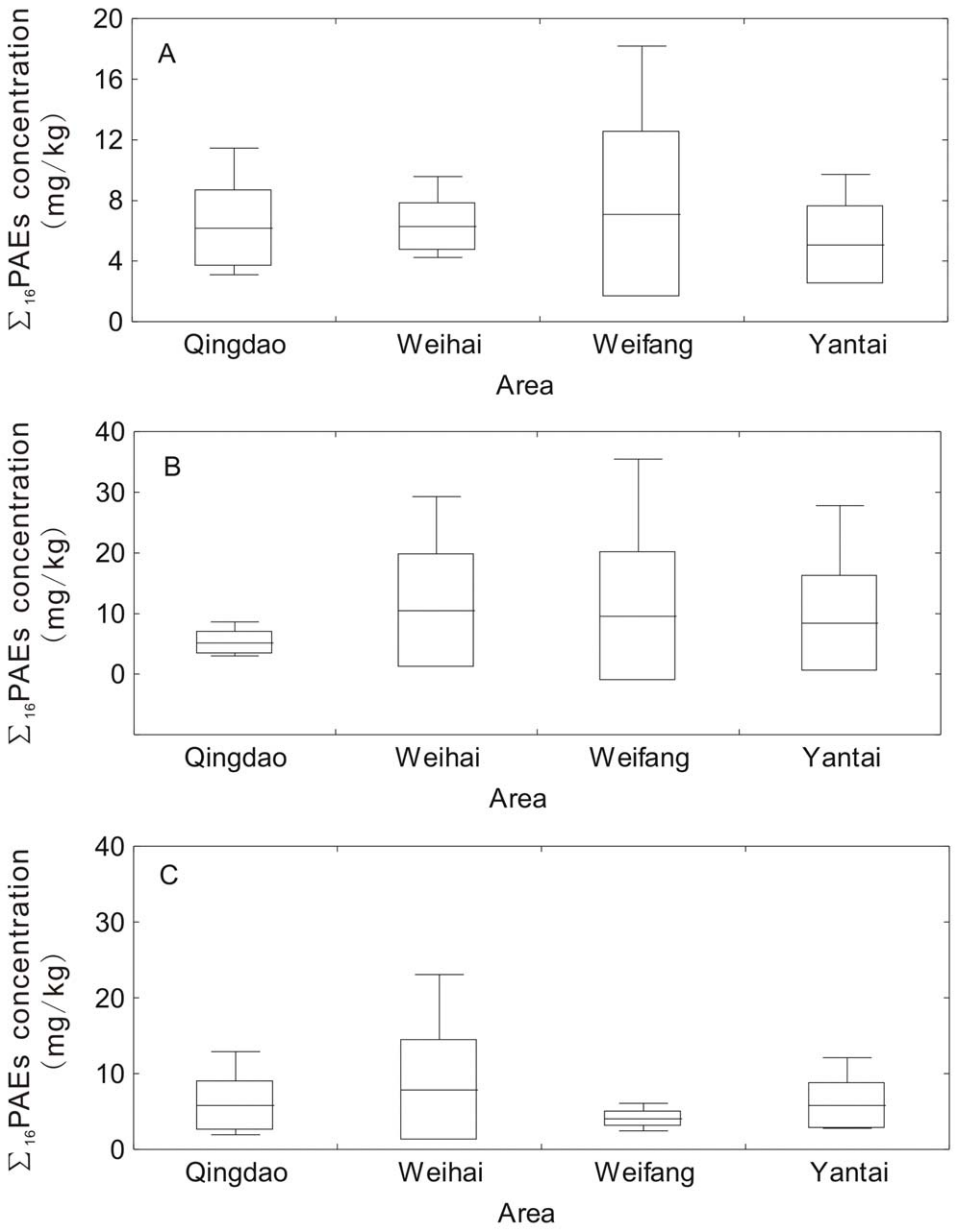
The concentrations of Σ_16_PAEs in (A) soils of 0–10 cm, (B) soils of 10–20 cm and (C) soils of 20–40 cm from vegetable greenhouses in Shandong Peninsula.

The concentrations of Σ_16_PAEs in soils from vegetable greenhouses in different areas of Shandong Peninsula are presented in Fig. 2. The variability of Σ_16_PAEs was high in Weifang in the two upper layers, but low in Qingdao (10 cm to 20 cm) and Weifang (20 cm to 40 cm). The maximum value of Σ_16_PAEs in soils at 0 cm to 10 cm and 10 cm to 20 cm both appeared in Weifang, with values of 18.179 and 35.442 mg/kg, respectively.

### PAE composition in soils of vegetable greenhouses from Shandong Peninsula

**Figure 3:**
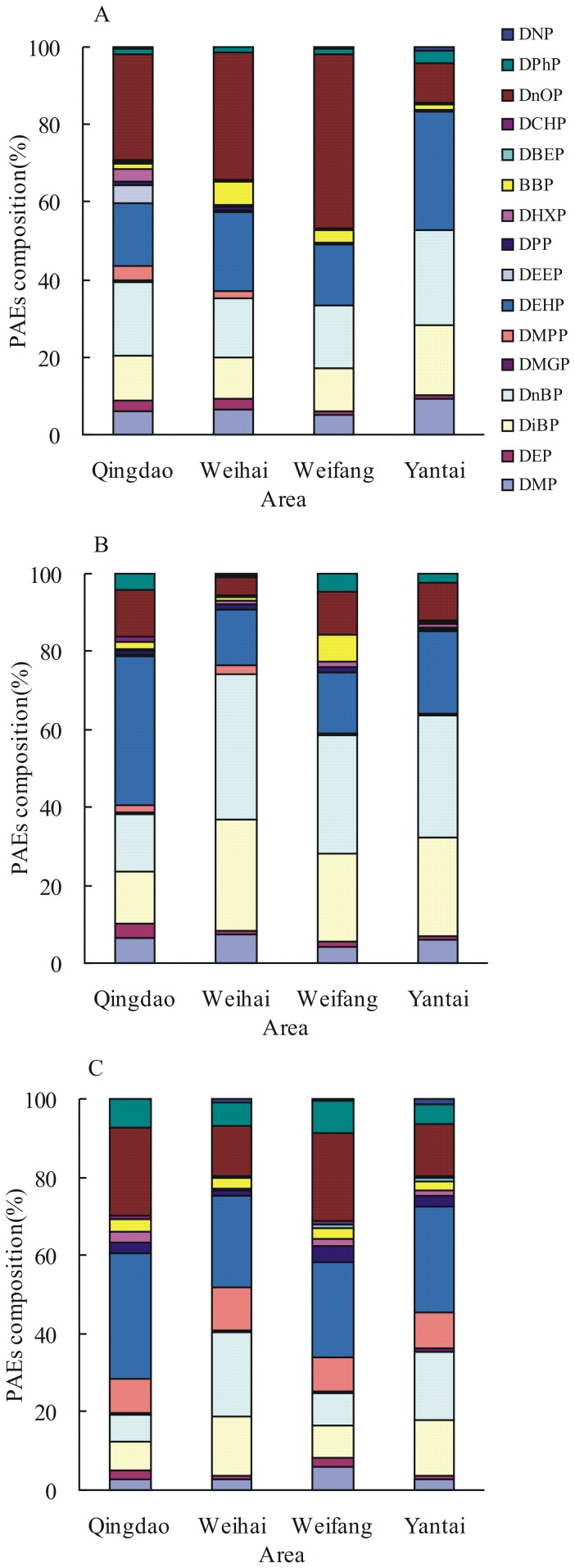
The compositions of PAEs in (A) soils of 0–10 cm, (B) soils of 10–20 cm and (C) soils of 20–40 cm from vegetable greenhouses in Shandong Peninsula.

Despite the different concentration of Σ_16_PAEs, the PAE compositions in soils from vegetable greenhouses in Shandong Peninsula were comparable (Fig. 3). DnOP had the highest proportion (27.1% to 45%) in soils at 0 cm to 10 cm in Qindao, Weihai, and Weifang, whereas DEHP had the highest proportion (30.4%) in Yantai. The proportion of DnBP and DiBP ranged from 10.6% to 24.5%, suggesting significant proportion. In soils at 10 cm to 20 cm, DEHP had the largest proportion of 38.4% in Qingdao, but DnBP had the largest and DiBP had the second largest proportion in the other three areas. In soils at 20 cm to 40 cm, DEHP was a dominant congener, ranging from 23.4% to 31.8% in four areas. DnOP in Qingdao and Weifang had the second highest proportion, whereas DnBP in Weihai and Yantai had the second highest proportion. Therefore, DEHP, DnOP, DnBP, and DiBP had the largest proportion in soils in all areas. In addition, DMP accounted for about 5∼10% in the upper two layers; by contrast, DEP, DMPP, BBP, and DPhP accounted for only approximately 1% to 5%, suggesting a small proportion.

## Discussion

### Potential sources of PAEs in soils from vegetable greenhouses

Soils that are being used or were used for vegetable greenhouses had higher PAE concentrations (Table 3), which suggests that PAEs are widespread in soils from vegetable greenhouses. Various PAE sources exist in soils from vegetable greenhouses. The plastic film used in vegetable greenhouses is a major source of PAEs. The maximum value of Σ_16_PAEs in soils at 0 cm to 10 cm and 10 cm to 20 cm appeared in Weifang (Fig. 2), which has a long history of vegetable production and is famous for extensive greenhouse cultivation. Even more remarkably, the plastic film used in greenhouse cultivation in Shandong Peninsula is replaced annually, which may result in a higher concentration of PAEs in soils from vegetable greenhouses than in other soils. In addition, PAEs are found in organic fertilizers in China [26] and in compost of sewage sludge with rice straw [27]. The amount of fertilizers used for vegetable planting in greenhouses is more than that used for field crops, and the proportion of organic fertilizers has increased since 2007 [28]. Moreover, PAEs are found in the groundwater in China [29]–[31], and groundwater is used for irrigation in vegetable greenhouse, which may result in the buildup of PAEs in vegetable greenhouse soils. More importantly, Zeng et al. [32] found a declining trend of PAEs in agricultural soils that were far from urban centers. The highest PAE contents are found in soils close to architectural markets, where plastic materials are sold, and those close to large chemical manufacturing factories. Most vegetable greenhouses in this study are near industrialized cities with large populations. Over 300 plastic manufacturers that produced 0.3 million tons/yr of plastic existed in Shandong Peninsula by 2003. All these factors may have resulted in the high concentration of PAEs in vegetable greenhouses in Shandong Peninsula.

**Table 3:** Comparisons of PAEs contents in agricultural soils in China (mg/kg).

Area	DMP	DEP	DnBP	DEHP	BBP	DnOP	Σ_6_PAEs	Σ_16_PAEs	Type of soils	References
Shandong Peninsula	0.36	0.11	1.47	1.47	0.19	1.24	4.48	6.73	Soils of vegetable greenhouses	In this study
	(ND∼1.24)	(ND∼1.21)	(0.02∼15.72)	(0.07∼5.32)	(ND∼5.69)	(ND∼14.40)	(1.18∼23.35)	(1.94∼35.44)		
Nanjing	0.006	0.005	0.19	1.72	0.003	0.158	1.89		Soils of vegetable greenhouses	[14]
	(ND∼0.016)	(ND∼0.012)	(ND∼1.41)	(0.034∼9.031)	(ND∼0.038)	(ND∼1.739)	(0.15∼9.68)			
Hangzhou	ND	0.59	0.21	1.48	0.05	0.14	2.47	2.75	Soils of vegetable greenhouses	[15]
		(0.06∼1.49)	(0.14∼0.35)	(0.81∼2.20)	(0.03∼0.16)	(0.10∼0.25)		(1.90∼4.36)		
								(Σ_11_PAEs)		
Guangzhou, Shenzhen,	(ND∼0.68)	(ND∼1.77)	(3.75∼18.45)	(2.82∼25.11)	(ND∼1.48)	(ND∼0.92)	(3.00∼45.67)		vegetable Soils	[16]
Zhangjiang,	0.02	0.09	0.23	0.15	0.05	0.03	0.56		Vegetable soil	[50]
Dongguan,	(ND∼0.45)	(ND∼1.06)	(ND∼7.65)	(ND∼6.38)	(ND∼2.83)	(ND∼0.32)	(0.01∼9.30)			
Zhongshan,	0.02	0.05	0.49	0.17	0.13	0.06	0.92		Paddy soil	
Zhuhai,	(ND∼0.86)	(ND∼1.60)	(ND∼17.51)	(ND∼4.22)	(ND∼5.89)	(ND∼1.12)	(0.01∼25.99)			
Shunde	0.03	0.19	0.41	0.12	0.02	0.02	0.81		Banana soil	
	(ND∼0.12)	(ND∼2.50)	(ND∼4.13)	(ND∼2.69)	(ND∼0.26)	(ND∼0.08)	(0.05∼5.92)			
	0.03	0.06	0.30	0.07	0.01	0.02	0.49		Sugarcane soil	
	(ND∼0.18)	(ND∼0.44)	(ND∼1.77)	(ND∼0.26)	(ND∼0.03)	(ND∼0.07)	(ND∼2.10)			
	0.02	0.06	0.20	0.11	0.01	0.02	0.43		Orchard soil	
	(ND∼0.06)	(ND∼0.40)	(ND∼0.56)	(ND∼0.99)	(ND∼0.06)	(ND∼0.15)	(0.04∼1.38)			
Leizhou Peninsula	0.02	0.02	0.45	0.01	0.01	0.02	0.53	1.11	Sugarcane soil	[17]
								(0.02∼5.45)		
	0.01	0.01	0.24	0.24	0.01	0.02	0.53	0.86	Paddy soil	
								(ND∼2.78)		
	0.02	0.01	0.27	0.12	0.01	0.02	0.45	0.61	Vegetable soil	
								(0.02∼1.87)		
	0.03	0.01	0.28	0.10	0.01	0.01	0.44	0.60	Orchard soil	
								(0.28∼1.05)		
Huizhou	0.004	0.01	0.15	0.09	0.002	0.01	0.31	0.60	Agricultural soil	[51]
	(ND∼0.03)	(ND∼0.22)	(ND∼0.39)	(ND∼0.44)	(ND∼0.04)	(ND∼0.06)	(0.09∼0.75)	(0.18∼2.04)		
							0.28	0.59	Vegetable soil	
							(0.06∼0.64)	(0.18∼2.04)		
							0.24	0.65	Paddy soil	
							(0.08∼0.64)	(0.43∼1.21)		
							0.22	0.51	Orchard soil	
							(0.08∼0.64)	(0.38∼0.63)		

Among 16 PAEs, DEHP and DnBP are found to be the two most abundant PAEs in agricultural soils in Guangzhou, Shenzhen, Leizhou Peninsula, and Huizhou (Table 3). Moreover, DiBP is found to be abundant in Guangzhou agricultural soils [32], whereas DnOP is abundant in the soils of vegetable greenhouses in Nanjing [14]. Similarly, DEHP and DnOP are the two most abundant PAEs in soils of vegetable greenhouses in Shandong Peninsula, followed by DnBP and DiBP (Fig. 3). The relative contribution of PAEs in agricultural soils is consistent with that in sediment [33], air [5], [34], and waters [35]. The global consumption of PAEs is about 6.0 million tones/yr, mainly as plasticizers in the plastic industry. Among plasticizers of PAEs, DEHP, DnOP, and DiBP/DnBP are widely used. It is found that DEHP and DnBP are two dominant PAE components in white and black mulch film used in vegetable production systems, ranging from 48.0∼115.6 mg/kg and 2.3∼3.2 mg/kg, respectively [14]. We also found that besides DEHP, DnOP and DiBP were two dominant PAEs in polyvinyl chloride (PVC) plastics mainly used in vegetable greenhouses in Shandong Peninsula, accounting for 20% and 10% of total of 16 PAEs, respectively. DMP and DEP are also detected in the plastics film, though their contents are low. Therefore, the plastics film may be a major potential source of some PAEs. Furthermore, PAEs are found in fertilizer and manure. DEHP, DnBP, DMP and DEP are the major organic pollutants in fertilizers, with contents more than 2.5 mg/kg [27]. Similarly, six PAEs (DEHP, DnBP, DnOP, DMP, DEP and BBP) are found in chicken, pig, cow and duck manure, in the range of 2.24∼6.84 mg/kg [14]. These potential sources may lead to the high detection rates of DMP, DEP DnBP, DiBP, DEHP and DnOP (Table 2).

### Relationship between PAEs and soil properties or age of vegetable greenhouses

Soil properties, such as pH, organic matter, texture, and redox potential, have a certain effect on the migration of hydrophobic organic compounds (HOCs) in soil [36]–[37]. A positive correlation between HOCs and TOC is found in several research [32], [38]; however, it is not in this study. Katsoyiannis [39] reported that no correlation can be found between HOCs and TOC if continuous sources of HOCs exist in soils. In this study, several continuous inputs of PAEs in vegetable greenhouse soils, including plastic film, fertilizers, and irrigation, may hinder the achievement of equilibrium between PAEs and TOCs.

**Figure 4:**
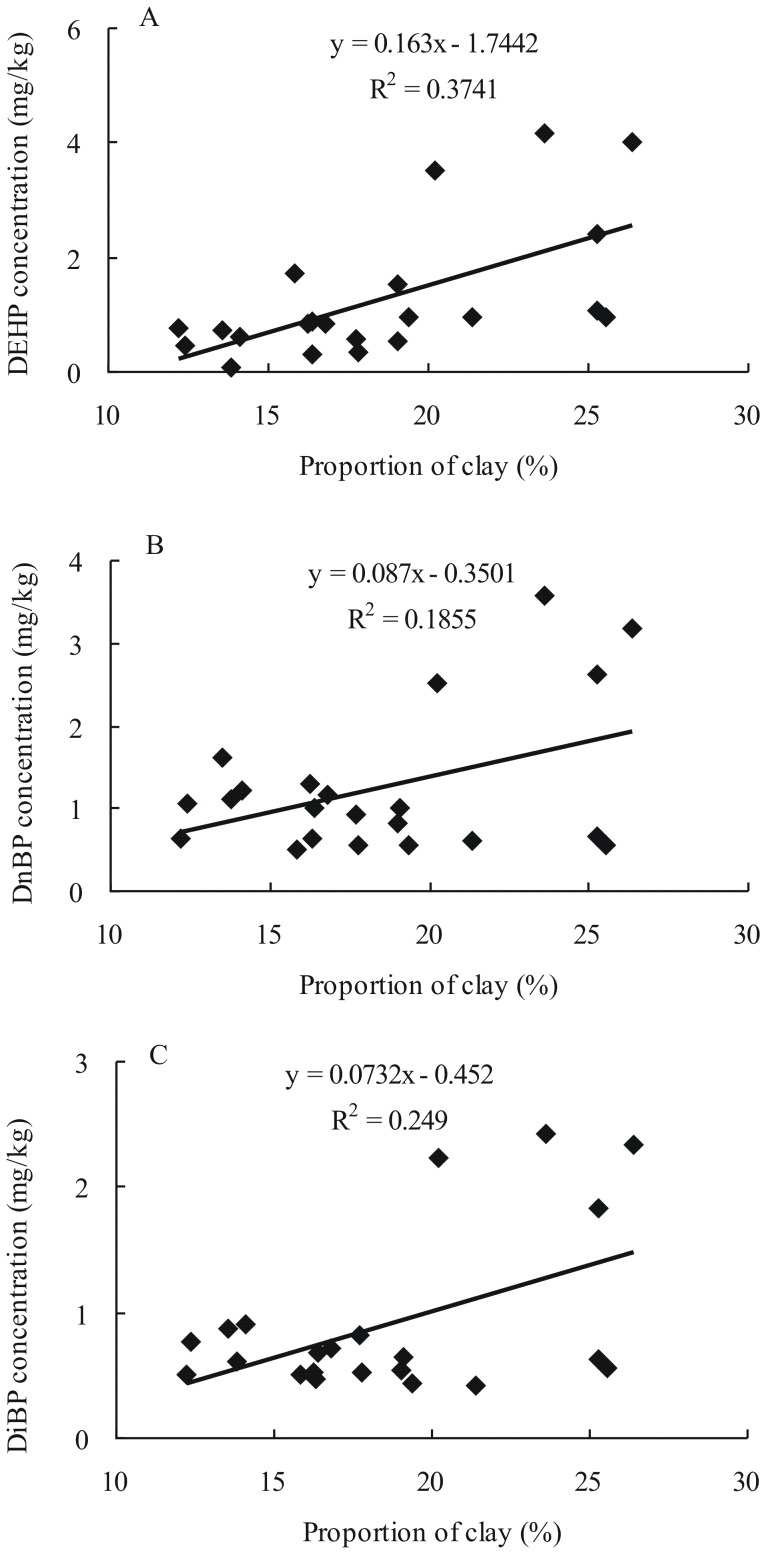
Correlations of the concentrations of (A) DEHP, (B) DnBP and (C) DiBP with the proportions of clay in 0–10 cm soils of vegetable greenhouses from Shandong Peninsula.

The relationship among the major PAEs, including DEHP, DnBP and DiBP, with the proportions of clay in 0 cm to 10 cm soils was analyzed (Fig. 4). DiBP, DnBP, and DEHP have a significantly positive correlation with the proportion of clay (*r* = 0.431∼0.611, *p*<0.05). A similar relationship of HOCs, such as PAEs, organic chlorinated pesticides, and PAHs, with clay is also found in soils [32], [40]–[41]. The clay of sediment or soil shows stronger capability to adsorb HOCs than sand and silt, due to small granulometry but high specific surface area [42]–[43]. Besides, the aging of organic matter, such as humic material, distributes around clay complexes, resulting in the formation of films of organic material [44]. These films of organic material are very difficult to remove, and so organic matter builds up and becomes a permanent part of the clay complexes. The clay-organic complexes supply rich reactive sites for the adsorption of organic pollutants [45].

A positive correlation between Σ_16_PAE concentration and age of vegetable greenhouses was found in this study (*r* = 0.294, *p*<0.01), suggesting that PAEs in soils may be related with the cumulative use of potential PAE sources over years in greenhouse vegetable cultivation. However, the correlation coefficient is not high. Studies demonstrate the biodegradability of some PAEs in aerobic condition [46]–[47], though PAEs are resistant to degradation through hydrolysis and photolysis. The biodegradability of PAE congeners is different. Shanker et al found the degradation rates of DMP and DBP were greater than that of DEHP under aerobic conditions [48]. Additionally, PAEs migrates deeper in soils profiles, and the TOC of soil and volume of leaching water can affect the migration of PAEs [49]. These factors may result in the low correlation coefficient between PAE contents and age of vegetable greenhouses.

### Comparison of PAE concentrations in the different soils in China

Comparisons of PAE contents in agricultural soils from China are presented in Table 3. The average Σ_6_PAE (DMP, DEP, DnBP, DEHP, BBP, and DnOP) contents in the soils from vegetable greenhouses in Shandong Peninsula, Nanjing, and Hangzhou are approximately 2 mg/kg to 6 mg/kg, higher than other types of soils. High Σ_6_PAE concentrations are also found in vegetable soils from Guangzhou and Shenzhen, where soils are previously used to plant greenhouse vegetables. In comparison, the Σ_6_PAE concentrations in vegetables, paddy, banana, sugarcane, or orchard soil are low, ranging from 0.2 mg/kg to 1 mg/kg.

### Potential risk assessment of soils of vegetable greenhouses from Shandong Peninsula

PAEs have a variety of toxic effects. Long term exposure to PAEs results in decreased fertility in females, fetal defect, altered hormone levels, uterine damage and male reproduction abnormalities such as reduced sperm production and motility, cell damage, cell tumors, etc [52]–[55]. According to human health based levels that correspond to excess lifetime cancer risks and human health based levels for systemic toxicant calculated from reference doses, allowable soil concentrations and cleanup levels of PAEs have been recommended in New York, USA [56]. The allowable soil concentrations are 0.02, 0.071, 0.081, 1.125, 4.35, and 1.20 mg/kg for DMP, DEP, DnBP, BBP, DEHP, and DnOP, respectively; and soil cleanup levels are 2, 7.1, 8.1, 50, 50, and 50 mg/kg, respectively [56]. PAEs exceeding allowable and cleanup concentrations may be a menace to human health. According to these criteria, the ratios of PAE concentration in this study exceeding allowable and cleanup concentrations are presented in Table 4. The ratios of DMP, DEP, and DnBP exceeding allowable concentrations are 90% to 100% at different soil depths, suggesting high PAE pollution. Moreover, the ratios of DnOP exceeding allowable concentrations are also high, particularly in soils at 0 cm to 10 cm; however, the ratios of BBP and DEHP are low. Similarly, in agricultural soils around Guangzhou, DMP, DEP, and DnBP also exceed allowable concentrations [32]. Notably, DnBP in some samples is approximately twice to thrice higher than the recommended cleanup concentration. These soil samples are mostly from the vegetable greenhouses with ages of approximately 10 years, suggesting that long-term application of plastic film or manure in vegetable greenhouses may increase environmental and health risks.

**Table 4:** Ratio of PAEs in samples exceeding allowable and cleanup concentrations in soils from vegetable greenhouses in Shandong Peninsula.

Soil depth (cm)	Ratio of samples exceeding allowable concentrations (%)						Ratio of samples exceeding cleanup concentrations (%)					
	DMP	DEP	DnBP	BBP	DEHP	DnOP	DMP	DEP	DnBP	BBP	DEHP	DnOP
0∼10	94.6	89.2	97.3	0	2.7	45.9	0	0	0	0	0	0
10∼20	100	94.6	94.6	2.7	5.4	27	0	0	13.5	0	0	0
20∼30	89.2	97.3	89.2	0	0	21.6	0	0	2.7	0	0	0

The cultivated vegetables can uptake and accumulate PAEs, but the difference is found in accumulated amount of PAE congeners by vegetables. Compared with DEHP, more DBP in soils is accumulated in stalk and leaf of carrot, cucumber and tomato [24]. The physical and chemical properties, such as molecular weight and octanol/water partition coefficient (*K*_ow_), have effects on the accumulation of PAEs by plants. Due to the smaller molecular weight and lower *K*_ow_, DBP is more easily absorbed and transported by vegetables than DEHP. Furthermore, several studies report a positive correlation between accumulated PAE amount by vegetables and contents in soils [24], [57]. Thus, mitigation of PAEs in soils is important to lower the risks of PAEs to human health.
